# Patient and public involvement in primary care research - an example of ensuring its sustainability

**DOI:** 10.1186/s40900-016-0015-1

**Published:** 2016-01-14

**Authors:** Clare Jinks, Pam Carter, Carol Rhodes, Robert Taylor, Roger Beech, Krysia Dziedzic, Steven Blackburn, Rhian Hughes, Bie Nio Ong

**Affiliations:** 1 Research Institute for Primary Care and Health Sciences, Keele University, Keele, ST5 5BG England; 2 Department of Health Sciences, College of Medicine, Biological Sciences and Psychology, University of Leicester, 22-28 Princess Road West, Leicester, LE1 6TP England; 3 Patient and Public Involvement Coordinator, Research User Group (RUG), Research Institute for Primary Care and Health Sciences, Keele University, Keele, ST5 5BG England; 4 Lay Representative, Research User Group (RUG), Research Institute for Primary Care and Health Sciences, Keele University, Keele, ST5 5BG England; 5 Keele University School of Nursing and Midwifery Clinical Education Centre, University Hospitals of North Midlands NHS Trust, Royal Stoke University Hospital, Newcastle Road, Stoke-on-Trent, ST4 6QG England

**Keywords:** Patient and public involvement, Impact, Sustainability of PPI, Primary care research, Resource allocation

## Abstract

**Background:**

The international literature on patient and public involvement (PPI) in research covers a wide range of issues, including active lay involvement throughout the research cycle; roles that patients/public can play; assessing impact of PPI and recommendations for good PPI practice. One area of investigation that is less developed is the sustainability and impact of PPI beyond involvement in time-limited research projects.

**Methods:**

This paper focuses on the issues of sustainability, the importance of institutional leadership and the creation of a robust infrastructure in order to achieve long-term and wide-ranging PPI in research strategy and programmes.

**Results:**

We use the case of a Primary Care Research Centre to provide a historical account of the evolution of PPI in the Centre and identified a number of key conceptual issues regarding infrastructure, resource allocation, working methods, roles and relationships.

**Conclusions:**

The paper concludes about the more general applicability of the Centre’s model for the long-term sustainability of PPI in research.

## Plain English Summary

We describe how a research centre has developed and sustained the involvement of patients and the public in its research. Patient and Public Involvement (PPI) in research is often organized for individual projects and can sometimes be ‘tokenistic’ where there are no relationships between researchers and PPI advisors, is minimal support for PPI and a lack of feedback or follow up. We highlight how a Research User Group was formed and is supported to ensure that lay people can be long-term partners in research. Key to the meaningful and long-term involvement of lay people is organizational commitment and leadership, adequate resourcing and dedicated support infrastructure.

## Background

Patient and Public Involvement (PPI) in research has been well documented. There are many examples of involvement throughout the research cycle, clarifying the roles that lay people may play and models of good practice [1]. Debates persist about the distinctions between ‘meaningful’ and ‘tokenistic’ involvement, and how to assess the impact of involvement. Two reviews of the impact of involvement found that PPI costs money and time [2, 3]. We set out our experience of PPI and demonstrate how academic theory about involvement was combined with practical and organizational learning, ensuring sustainability of PPI over time. Our theoretical approach is influenced by studies of public involvement in research that draw on concepts such as participatory democracy, deliberation where people are given information and an opportunity to consider this, leading to discussion and dialogue [4, 5]. Combining theory and reflective practice resulted in positive gains for the organization and for the lay people involved.

The importance of planning for sustainable involvement has been previously recognized [6]. One example of longer-term involvement is OMERACT (Outcome Measures in Rheumatology) where rheumatology patients have worked with clinical researchers in a series of conferences [5]. Howe et al (2010) have also reported on long-term resourcing and embedding of PPI in joint university and NHS research [7]. Yet, knowledge about a structural approach to sustaining PPI in primary care research remains limited and guidance tends to focus on involvement at the project, rather than programme or organizational level [8]. We discuss the requirements necessary to ensure that lay people can be long-term partners in primary care research, focusing on organizational commitment and leadership, adequate resourcing and building a support infrastructure.

### The setting: a primary care research centre

Primary care research is faced with a number of specific challenges. Patients may have acute or chronic conditions and many patients have two or more chronic conditions (multimorbidity). Management of chronic conditions and multimorbidities is a key feature of primary care practice and can be complex. Patients may have long term relationships with health care practitioners and long term experience of using health services. Musculoskeletal conditions such as osteoarthritis are not seen as a priority for many health care practitioners.

Therefore, it is important to obtain input from primary care patients to ensure that the research agenda and process is relevant to their needs.

The Centre involved lay people from the late 1990s in a few discrete research projects, but set up a Research User Group (RUG) in 2006 with the aim to embed PPI across the Centre’s work. RUG members were recruited from previous projects and 12 people suffering from a range of conditions, but primarily musculoskeletal pain, started in August that year. A senior researcher led the RUG with administrative support, and funding was earmarked by the Centre’s Co-Director. Activities included discussing new ideas with researchers, taking part in project and steering groups, presentations to funders and holding a national conference. By 2009 the Centre’s portfolio had grown and so the need for more lay people became apparent and the RUG was expanded with a further 19 people. The day-to-day organization of PPI could also not be continued part-time by senior researchers and a PPI User Support Worker was appointed. This individual was someone who herself suffered from chronic widespread pain, and thus possessed shared illness experiences with the RUG members. The work expanded to include national and international conferences and exchange alongside continued involvement on large Centre research programmes and projects. RUG members requested more training and support, and in turn also trained researchers in how to effectively involve lay people in their research. By 2012 the PPI User Support Worker was promoted to PPI Coordinator and was joined by a new Support Worker (who is also a patient) as the organization of all the RUG activities was becoming increasingly complex. In the autumn of 2012 a further recruitment drive was launched as the Centre’s remit widened to include long-term conditions and mental health. Currently, 68 lay people work alongside researchers in 14 research groups. 

## Methods 

In 2010-2011 an evaluation was conducted to better understand the nature of PPI at the Centre and learn lessons for improvement. The evaluation consisted of face to face interviews with 17 Centre staff and 15 RUG members, and an analysis of documentation (*n* = 90 documents, including minutes of meetings, agendas, reports, presentations and magazine articles). The interviews were analysed using an analytical framework, with themes drawn from literature [1–3]. The quotations from researcher and RUG members in this article are taken from the final (internal) report. Ethical approval was given by Keele University ethics committee in 2010.

## Results and discussion

### Sustaining involvement: operating real partnership and structuring support

The members of the RUG are recruited on the basis of their illness experience – rather than educational attainment or prior research involvement – and thus they bring this ‘expertise by experience’ to the table and are generally interested in research. On joining the RUG, members provide brief details about themselves (e.g. age, health condition, medication use, work status) which are stored on a database. The PPI coordinator and user support worker then use this information along with personal knowledge about individual members, regarding their experiences, interests and previous/current involvement in studies, to search and match suitable RUG members to the requirements for PPI in individual studies. Selecting the right people for involvement in studies is challenging and is not always successful. However, essential to this process is the friendly relationship and trust between the PPI staff and RUG members, alongside a systematic method of storing and retrieving information about RUG members.

On the other hand RUG members ‘life world’ comes into contact with the ‘system world’ of academic research [5] which may cause tensions. Good practice guidance notes the importance of avoiding a ‘hit and run’ approach whereby researchers engage in a minimal fashion with patients or service users. Building mutual understanding and respect takes time, not only within the actual research meetings, but establishing trust does require long-term investment from both parties. The distinction between the ‘tick box’ approach and the importance of genuine investment is exemplified in what a senior manager said:

“I think it’s been an interesting journey for, for the Centre because I think that, you know, I think the users are very much valued, you know, and I, and I suspect, even, even if it did start that this is something we’d better do, I think we’ve moved on a long way from that now and I think the users are, are really valued.”

In order to facilitate the creation of effective partnerships a number of strategies were put into place:

1. PPI meetings were designed to be informal but to focus on the task at hand
2. Provision of dedicated support for RUG members to contribute to research (e.g. support worker)
3. Logistical support (organizing accessible car parking and venues, etc)
4. Avoidance of research jargon and use of plain English.
5. Provision of training and guidance for RUG members
6. Recognition of the need for timely feedback to RUG members

These strategies are discussed further below.

The conduct of meetings required balancing informality and allowing space for patient experiences to be aired while maintaining a focus on the work. Thus, the RUG members grew to appreciate that the RUG was not a mutual support group, but that their experiences were valuable in terms of shaping the research questions, design and operationalization. This also led to a more formalized organization of meetings as one of the RUG members explained:

“When we started, the meetings were very ad hoc. It depended on what they were doing and if they needed us, so to speak. And I think we’re going towards, or have gone towards, the fact that it would be better if we had a regular two-monthly meeting.” (U11)

RUG members needed dedicated support in carrying out a range of tasks. For example, the User Support Worker prepared them for Steering Group meetings and often accompanied individuals. Conversely, the PPI coordinator met with researchers to ensure that their presentations to the RUG, or supporting documentation, were appropriately targeted and not jargonized. The PPI coordinator role was recognized by senior staff as essential:

“I think [PPI Co-ordinator] is helping the user group to enable them to be a little bit more, not critical exactly, but perhaps a little bit more assertive, in terms of asking, ”Why are you looking at that, isn’t this just as important as well?” (S14)

Logistical support was of great importance: organizing car parking, accessible venues, booking train tickets and hotels (for external meetings or conference attendance). The RUG members considered this aspect as helping them to reduce worries:

“The parking here is a wonderful asset, to have parking easy here, not just the disabled badge, but having the parking sorted for everybody solves a major anxiety I think that’s fair to say.” (U4).

Scientific language can be intimidating and the PPI coordinator, with support from researchers, developed a glossary of terms and a series of leaflets for new RUG members to help them navigate research terminology and processes. The metaphor of speaking a foreign language was used to describe the difference between lay people and researchers:

“I just think of it, it’s like, if you put me in a room of people talking French how, how can I contribute? But if you give me some terms then I could tell you what I wanted to eat or what I wanted to drink. And that’s the same with the users isn’t it? They don’t need to know everything.” (SC1)

RUG members increasingly recognized the need for training and this was put into place, responding to specific needs, such as assertiveness training (to optimize their input at meetings with professionals), explanation of different research designs, understanding systematic reviews or sessions with clinicians about particular conditions and the latest treatments. The balance between remaining close to the lay experience and becoming an ‘expert’ was debated, but in general a pragmatic approach was adopted as one of the senior researchers explained:

“I think it depends, I suspect one size doesn’t fit all, so, for example, to sit on the trials steering committee, I think it would be cruel to send somebody in without some training. So I think from that perspective, erm, people would need, would need some training, and I think they need to be able to communicate and they need to be reasonably confident that they can speak out and not feel intimidated by the group.” (S13)

The importance of feedback to RUG members on the outcomes of involvement was recognized early on. Hence summaries of meetings (and decisions made) or outcomes of grant applications were routinely sent to those who had been involved. An example was given by one RUG member appreciating that she received specific feedback from a GP researcher:

“…that was, that was very good for me because it made me feel that all the thinking I’ve done about it, you know, because I’d given it some thought and somebody put some ideas in me head, and again you see I had to jot those down before I went [laughs] because I didn’t know they would bring them up. And you get lost in the meetings.” (U10)

Putting into place structured support and ways of working that made RUG members feel valued and respected meant that a stable and well-informed group of lay people was created and could be sustained. Of the original 12 RUG members 6 are still active (one person died and 5 withdrew because of health/personal reasons), 11 of the people recruited in 2009 and 24 who joined in 2012 have remained involved to date. Along with new members joining since 2012, we currently have 68 RUG members. Some are more active than others across several studies, some members are involved in single studies and others are currently unavailable due to personal choice or illness or are not involved in a current study.

### Sustaining involvement: organizational leadership

**Fig. 1:**
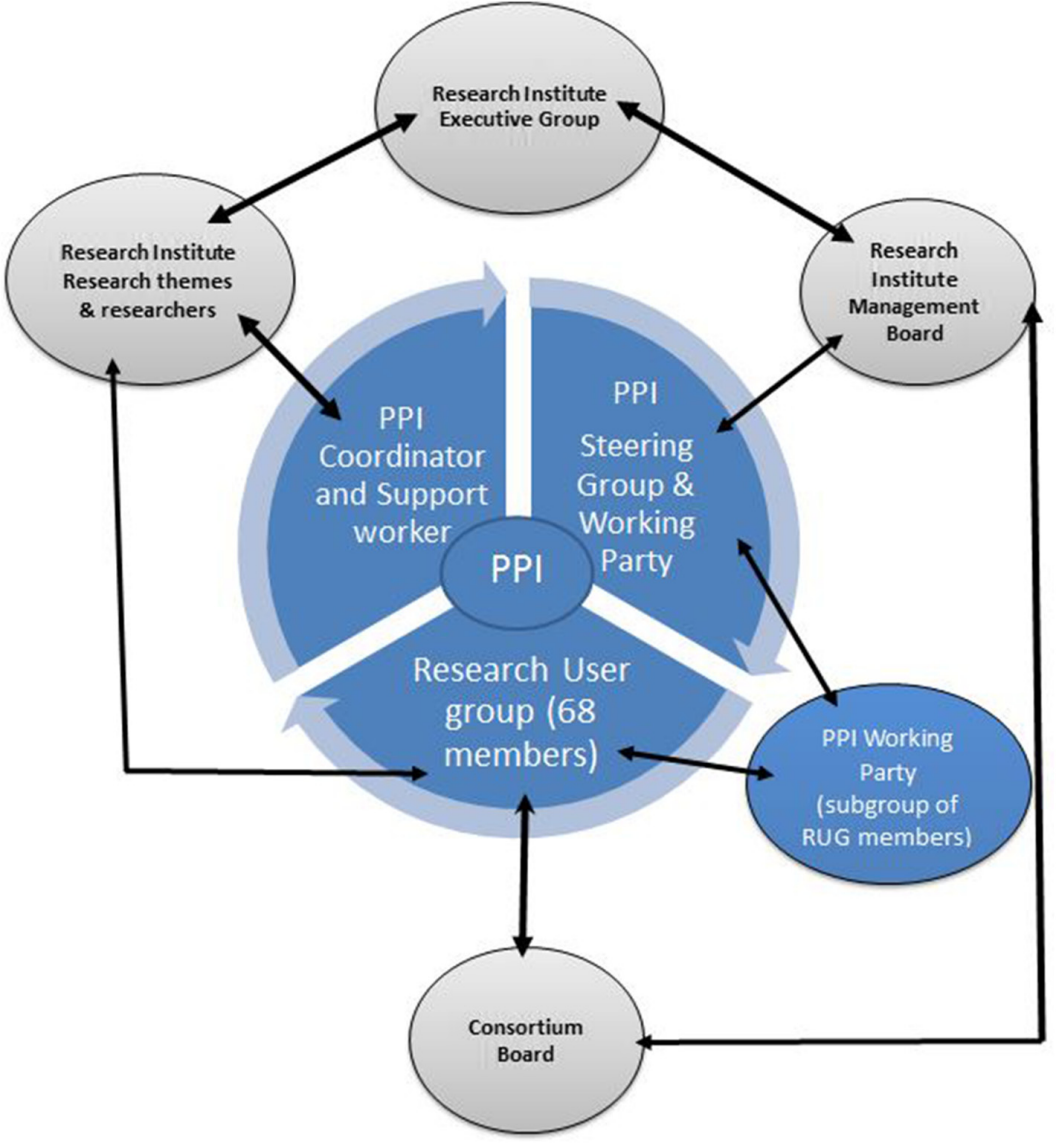
The organisational structure of patient and public involvement in the Primary Care Research Centre

From its inception the RUG has been chaired by a senior academic and this was considered important from the perspective of the RUG members: they saw this as symbolic of the Centre’s commitment. For the Centre it meant that PPI was highlighted at the strategic level and represented within senior management [9]. Over time this has become more formalized within the Institute Management Board (the Centre is part of a larger Research Institute (RI)) and with RUG representation on a Local Consortium Board (that oversees research between NHS, primary care and the university). Thus, PPI is fully embedded in organizational structures and PPI leadership remains part of senior academics’ roles. This is operationalized through joint membership of the Institute’s Management Board and Research Steering Committees (both which have links with the RI’s Executive Group) alongside membership of a PPI steering group (consisting of the PPI coordinator, support worker and PPI research leads) and its task-focused PPI Working Party. This structure is presented in Fig. 1. This figure also illustrates the core PPI functions and how researchers have access to the RUG.

This academic leadership is equally important for research staff as they realize that PPI is not optional, but an integral part of the way the Centre operates. Thus, new researchers learn about PPI as part of their induction. All research teams know that building in PPI from the start of any proposal is required and they meet with the PPI coordinator to plan and match the RUG members with the requisite skills to their proposals and funded work. The PPI team and senior academics supporting them ensure that PPI remains an organizational priority, for example, with the Centre gaining Clinical Trials Unit status, PPI has been formally included within its systems and operating procedures.

Another significant development has been the inclusion of RUG members in a communications and dissemination group (which reports to the Institute’s Management Board) as their input into making the research results relevant to patients and the public is invaluable. Furthermore, a subset of ten RUG members have formed a RUG Working Party to represent the the whole Research User Group within the Centre, be the focal point PPI in the Centre’s work and provide an arena for discussion with management on PPI issues and Centre Strategy.

**Fig. 2:**
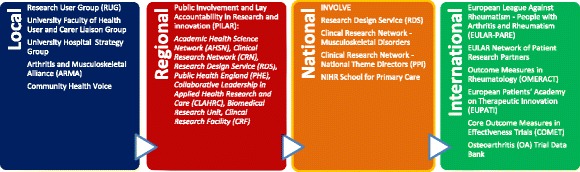
Key links with other organisations dedicated to patient and public involvement in research

Beyond the Centre, the academic leadership has stimulated wider recognition, for example, through ensuring that the PPI team and/or RUG members are invited to (inter)national meetings, and asked to present the unique way in which they have secured long-term involvement. Furthermore, this external presence has established key links with other organizations and enabled involvement in networks dedicated to patient and public involvement in research (Fig. 2).

### Sustaining involvement: resourcing PPI

Rewarding PPI contributions is known to be complex, especially for people in receipt of state benefits. Tackling this complexity demonstrates organizational commitment. The costs associated with PPI are often not clear, and the Centre is currently leading a national collaboration to investigate the costs and consequences of PPI in primary care research (funded by the NIHR School for Primary Care Research). The Centre has tried to formulate a transparent approach to costing, led by the Co-Director, and supported by the Primary Care Research Consortium and Arthritis Research UK. Following INVOLVE guidance RUG members are not only reimbursed for travel expenses, but also for replacement care if they have responsibilities for dependent relatives. The Centre developed a reward and recognition policy with advice from the Citizen’s Advice Bureau, and the system is user-friendly.

This approach has helped to provide realistic costings of PPI in research proposals. Apart from direct payments to RUG members, other costs are calculated such as training, workshop/conference presentations [10] and attendance, exchange visits with other PPI teams, production of materials and specific adaptations. The salary costs for the PPI team are also proportionally allocated, as is senior researcher support. We suggest that this is important for other research centres that are not necessarily as well resourced, in order to build up research infrastructure and create a critical mass of PPI expertise. In England Public Involvement Funds are available from the National Institute for Health Research, Research Design Service (RDS). However, we recognize that much of the funding available is linked to individual research projects, not for infrastructure purposes. In our Centre, PPI costs for two staff posts in the last five years, have totaled £199,165 (£39,833 per year for the last 5 years). Payments and expenses for RUG members in the year 2012/13 were £2555 and in 2014/15 were £2608.

The financial commitment by the Centre and its funders has been crucial in creating a robust foundation for all PPI work. Not only does it allow the PPI structure to be integrated within the Centre and a stable, dedicated team to be created, it also directly funds RUG members and make them feel that their contribution has value because their time and effort is being recognized. It is important to stress that most people are involved because of their interest, rather than because of the financial benefit. The words of this RUG member reflect this sentiment:

“I didn’t realise in the first place that at some of them you get paid for, I didn’t realise that, you know, so erm … I sat at the first meeting and somebody came in and said, ‘You’re getting 40p a mile for travelling,’ right okay, and you’ll get da, da, da, I think it was £170 a day for a full day or pro rata of that. I thought, ‘Wow’, you know. But erm that wasn’t the reason for doing it really, so that was a bonus, yeah.” (U5).

**Fig. 3:**
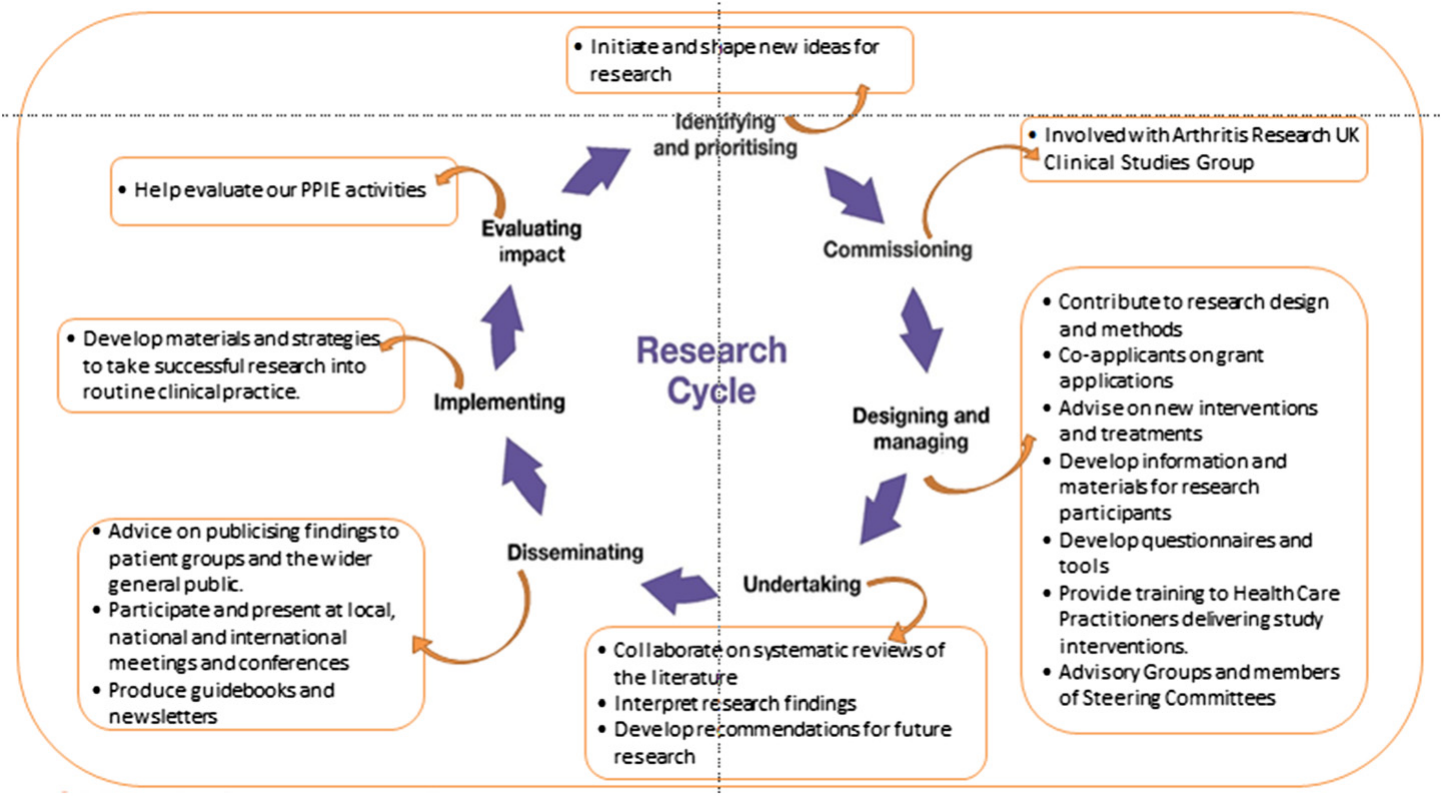
Examples of the impact of patient and public involvement in research conducted at the Primary Care Research Centre

Our emphasis on infrastructure has been deliberate as this tends to be insufficiently emphasized as a necessary condition for embedding and sustaining PPI. Securing this stable base facilitates the genuine involvement in all stages of the research process as exemplified in Fig. 3.

### Organizational learning

The evaluation of PPI in the Centre found that positive impacts of PPI for RUG members included enhanced skills, confidence building, keeping active in retirement, increased self-confidence and social support. Some negative impacts were fatigue, and stress/worry, for example, one RUG member with fibromyalgia said:

“…the main thing is the chronic fatigue and it’s a major thing you know. And I do think that it needs taking into consideration. As does travelling, you know. Going to London when you’ve never done anything like that before.” (U10)

Researchers mentioned positive impacts such as personal satisfaction and encouragement, while some perceived a threat to professionalism and the additional time taken up by PPI processes. Study teams reported enhanced ethical practice and improved validity of research instruments. The ability to test out the feasibility of study designs means that researchers can be more confident that their study designs are viable in the “real world” outside of the University. Finally, the evaluation found that PPI made a difference to funders and external visitors to the Centre who were impressed by RUG members’ ability to speak “from the heart”. A senior researcher summarized this as follows:

“And I think it’s also, it’s also helpful when we have external visitors, or on the study steering groups, when there are external people to have the users or the patients giving a very positive message about the research is really helpful.” (S13)

General acceptance of PPI by researchers is not always straightforward, and some consider PPI insufficiently important, limit involvement to certain parts of the research process or engage in ‘tokenistic’ engagement. The main strategy from those leading the PPI programme has been to demonstrate the benefits of PPI as mentioned earlier. Furthermore, there have been challenges for researchers to ensure good practice, specifically:

- ensuring RUG members receive timely feedback on the contribution to studies
- clarifying the roles of lay co-applicants
- ensuring meaningful contribution of RUG members on projects steering, and committees
- managing the differing expectations for RUG members.

Funding calls with short submission deadlines can sometimes cause difficulties. Balancing the needs of researchers for meaningful PPI in grant applications, with ensuring that RUG members *can* make a meaningful contribution without overburdening or pressuring them in a short time period is complex. These issues can cause RUG members to be very frustrated, though many have continued their support for the Centre. RUG members are also not homogeneous and have different levels of commitment, experience and skills. While these challenges continue, the Centre’s PPI team are working closely with the RUG and researchers, listening to their views and experiences of involvement and continuing to review procedures.

### The perspectives of the lay co-authors on sustaining PPI

Establishing a sustainable infrastructure and support system will help to maintain the long term interest and involvement of RUG members. It creates an environment where RUG members can feel ownership and pride in the studies they are involved in, and helps build long-lasting and effective working relationships with researchers. For example, a RUG member and co-author of this manuscript (RT) commented:

“As a member of the Research User Group for over 3 years I can fully support the views expressed in this paper. The Centre’s PPI model has created a sense of community and ongoing enthusiasm within our group, recognising and respecting our views at all times. This sense of mutual respect and working in partnership with researchers is key in my view in sustaining the continued interest of our members.”

The PPI Coordinator (and co-author CR) summarised the perspective of the RUG group on their long term involvement with the Centre’s research as follows:

“For health research to make a difference to the general public, the voice of the patient needs to be heard throughout the whole research process. I have felt privileged as a PPI Coordinator to work within an organisation that was forward thinking, providing the resources needed to make this happen – both paid roles for support staff and allocation of researchers’ time to help RUG members understand research processes such as ethics, systematic reviews and statistical results. The development of these relationships between staff and RUG members has helped to embed PPI as a sustainable and integral part of the Centre.”

Since completing our evaluation the RAPPORT study [11] has been published which outlines six salient actions that are required for positive outcomes and impact of PPI. Assessing how the Centre compares, we conclude the following:

1. The researchers and lay representatives having a shared understanding of the moral and methodological purpose of PPI: through the terms of reference for participants in the RUG and other structures both parties are clear about roles, responsibilities and the purpose of involvement.
2. Having a key individual coordinating PPI, with the PPI team as a whole also fulfilling this function.
3. Lay representatives having a strong connection with the target study population: all lay members have relevant illness experiences, and many are members of condition-specific support groups or patient participation groups.
4. The whole research team being positive about PPI input and fully engaged with it: in general Centre researchers support PPI, but this needs to be continuously worked on and reinforced.
5. Efforts to develop relationships established and maintained over time: we have demonstrated the considerable time commitment that has been sustained since 2006.
6. PPI is evaluated in a proactive and systematic approach: thus far one independent evaluation has been carried out, and the Centre has been part of the cost and consequences study funded by the NIHR School for Primary Care Research [12].

## Conclusion

The argument by Baggott and colleagues [13] is relevant where they note that when involving people with long term conditions trust between patients and clinicians can develop over time. This has its parallel in the involvement of lay people in health research, and sustaining PPI in primary care research requires a multi-pronged strategic approach. For the Centre this has meant the commitment from the organizational leadership to implement a holistic and embedded model. Finally, ongoing evaluation of PPI allows regular review of “fitness for purpose” so that arrangements can be adjusted according to strategic changes. Ensuring a consistent approach to achieve meaningful PPI across a research centre with over 55 studies currently being delivered and 13 studies being developed requires an appropriate governance and organizational strategy for PPI. This results in a upscaling in planning and resourcing which sets it apart from PPI in time limited individual studies. Embracing PPI throughout the governance and operational levels of the research centre helps to create a culture that PPI is an essential aspect of research. This encourages high quality, appropriate and meaningful PPI in all of it studies. Sustaining PPI in this context requires:

1. Strong and genuine academic leadership, alongside RUG representation within the governance structure of the research centre, to ensure that lay people are fully supported and feel valued, and to maintain awareness amongst researchers of the importance of PPI in their work.
2. Clear organizational commitment in terms of appropriate resourcing of PPI through its core funding and including realistic costings in all research proposals.
3. Creating a PPI infrastructure with dedicated staff to support lay people and to work with researchers in order to optimize effective relationships with those individuals.

We cannot emphasise enough that the time taken to build trusting relationships is considerable: to prepare for meetings and joint presentations, to give practical and emotional support, and to provide continuous feedback. Finally, the consistent and visible commitment from senior academics is key to building lasting relationships.

## Abbreviations

GP: general practitioner
NHS: National Health Service
OMERACT: Outcome Measures in Rheumatology
PPI: patient and public involvement
RUG: Research User Group
